# The clinical significance of plasma clusterin and Aβ in the longitudinal follow-up of patients with Alzheimer’s disease

**DOI:** 10.1186/s13195-017-0319-x

**Published:** 2017-11-23

**Authors:** Jung-Lung Hsu, Wei-Ju Lee, Yi-Chu Liao, Shuu-Jiun Wang, Jong-Ling Fuh

**Affiliations:** 1 Graduate Institute of Humanities in Medicine, Taipei Medical University, Taipei, Taiwan; 2 Department of Neurology, Chang Gung Memorial Hospital Linkou Medical Center and College of Medicine, Chang-Gung University, Taoyuan, Taiwan; 3 Taipei Medical University Research Center for Brain and Consciousness, Shuang-Ho Hospital, New Taipei City, Taiwan; 4 Neurological Institute, Taichung Veterans General Hospital, Taichung, Taiwan; 5 Faculty of Medicine, National Yang-Ming University School of Medicine, Taipei, Taiwan; 6 Institute of Clinical Medicine, National Yang-Ming University School of Medicine, Taipei, Taiwan; 7 Brain Research Center, National Yang-Ming University School of Medicine, Taipei, Taiwan; 8 Department of Neurology, Neurological Institute, Taipei Veterans General Hospital, Taipei, 112 Taiwan

**Keywords:** Clusterin, Alzheimer’s disease, Predictor, Progression

## Abstract

**Background:**

Clusterin and beta-amyloid (Aβ) are involved in the pathogenesis of Alzheimer’s disease (AD). The clinical significance of plasma clusterin and Aβ in AD progression remains controversial.

**Methods:**

We recruited 322 patients with AD and 88 controls between August 2012 and June 2013. All participants were evaluated at baseline with a clinical assessment, Mini-Mental State Examination (MMSE), and Clinical Dementia Rating (CDR) scales. Patients with AD were evaluated annually with the MMSE and Neuropsychiatric Inventory (NPI) scale during the 2-year follow-up period. The levels of plasma clusterin, Aβ1–40, and Aβ1–42 at baseline were analyzed to study the longitudinal changes in the patient scores on the MMSE and NPI during the follow-up period.

**Results:**

Patients in the highest tertile of plasma clusterin levels showed significantly lower MMSE scores than those in the lowest tertile (*p* = 0.04). After adjustment for multiple covariates using the generalized estimating equation analysis, there was a significant decrease in the MMSE scores over the 2-year follow-up period among AD patients in the highest tertile of plasma clusterin levels compared with those in the lowest tertile (−2.09, 95% confidence interval (CI) = −3.67 to −0.51, *p* = 0.01). In apolipoprotein E (ApoE)4-positive AD patients, baseline measurements of the ratio of plasma Aβ1–42/Aβ1–40 in the highest tertile predicted an increase in NPI agitation/aggression scores over the 2-year follow-up period (6.06, 95% CI = 1.20–10.62, *p* = 0.02).

**Conclusions:**

Plasma clusterin could serve as a biomarker for the severity of cognitive decline. Plasma Aβ in ApoE4-positive AD could predict long-term agitation/aggression symptoms.

## Background

Alzheimer’s disease (AD) is the most common neurodegenerative dementia. It is characterized by a progressive loss of memory and other cognitive functions, and ultimately leads to total dependence. The early diagnosis and prediction of prognosis in AD is crucial but often very difficult in the early stages of the disease. In addition, 75–90% of AD patients develop “non-cognitive” behaviors, affect/mood, and psychosis symptoms that are collectively described as behaviors and psychological symptoms of dementia (BPSD). BPSD are classified into various clusters using the Neuropsychiatric Inventory (NPI) [1–3]. Two biomarkers, amyloid beta peptide (Aβ) and clusterin, are associated with AD-related phenotypes [4, 5]. Previous reports have shown significant changes in the plasma levels of Aβ in patients with AD [6, 7], and a decrease in the plasma Aβ1–42/Aβ1–40 ratio was inversely correlated with the neocortical amyloid burden, which is the key pathogenic process in AD. However, other studies have not detected an association between the plasma Aβ levels and AD [8]. This controversy about the relationship between the plasma Aβ levels and AD may be the result of using different methods to measure Aβ and other factors that may interfere with the detection of Aβ, such as albumin and transthyretin [9, 10]. Longitudinal studies have reported both positive and negative associations between plasma Aβ levels and cognitive decline, and the long-term clinical role of plasma Aβ levels remains to be determined [4, 11].

Clusterin, also known as apolipoprotein-J, is a multifunctional lipoprotein involved in Aβ fibrillation, clearance, and complement inhibition [12, 13]. Clusterin is expressed in many tissues, and its expression in the brain is increased in AD [14, 15]. In AD patients, clusterin co-localizes with dystrophic neurites as well as Aβ in amyloid plaques [16]. Studies of cerebrospinal fluid (CSF) have found both elevated and decreased levels of clusterin in AD patients [17, 18]. It remains to be determined whether the CSF clusterin levels can be used for the diagnosis of AD. More consistent data were derived from plasma studies. Elevated plasma clusterin levels in AD are correlated with AD pathology, prevalence, and severity; therefore, elevated clusterin levels have been suggested as a peripheral signature of AD [19–21]. One longitudinal study suggested that the plasma clusterin level was a good candidate marker for disease progression in AD [5]. Some studies of patients with mild cognitive impairment (MCI) or AD showed that elevated levels of plasma clusterin were associated with rapid cognitive decline, whereas other studies reported the reverse [22, 23].

Previous studies on prognostic factors of AD identified the age of onset, years of education, and disease severity at baseline as independent predictors of rapid cognitive decline [24, 25]. While the plasma clusterin level may or may not predict cognitive changes in AD, the clinical importance of plasma Aβ levels over time remains unknown. In BPSD, early executive dysfunction may be a predictor of subsequent behavior disturbance [26]. There is nothing known about the association between plasma biomarkers and BPSD. Plasma Aβ may associate with clusterin as part of a clearance mechanism and contribute to other symptoms of AD over time [13]. In this study, we explored the clinical significance of plasma clusterin and Aβ during a longitudinal study of patients with AD.

## Methods

### Participants

We recruited patients with AD from the Neurology Department of Taipei Veterans General Hospital and the Taichung Veterans General Hospital in Taiwan between August 2012 and June 2014. Subjects who were between 60 and 90 years of age, of both genders, and had adequate visual and auditory abilities to perform all aspects of the study assessments were included. Diagnoses of AD were made by consensus during a multidisciplinary meeting based on the clinical criteria for probable AD as described by the National Institute on Aging—Alzheimer’s Association [27]. The exclusion criteria included any significant neurological disease other than AD that might affect cognition, including Parkinson’s disease, multi-infarct dementia, Huntington’s disease, normal pressure hydrocephalus, brain tumors, progressive supranuclear palsy, seizure disorders, subdural hematoma, multiple sclerosis, a history of significant head trauma followed by persistent neurologic defaults, or known structural brain abnormalities. The informant who received the examinations spent at least 10 h per week with the patient and was knowledgeable about their recent and past medical history. Controls were recruited from the spouses of AD patients included in the study and cognitively healthy patients at the Department of Neurology for the assessment of other disorders. Patients with a history of major neurological, psychiatric, or severe cardiovascular diseases were excluded. All participants received a standardized assessment that included a clinical interview, neuropsychological assessment, laboratory tests, and brain magnetic resonance imaging (MRI). Age, gender, body mass index (BMI), years of education, and disease duration were recorded for demographic analysis. Disease duration was defined as the onset of symptoms (as reported by the informant) until this study was performed. All information on patients and controls is summarized in Table 1. The Institutional Review Board of Taipei Veterans General Hospital approved this study (IRB number: 2012-05-033B). Written informed consent was obtained from all participants and their next of kin or legally authorized representatives.

**Table 1:** Demographic and clinical data of patients with AD and controls

	AD baseline (*n* = 322)	AD 1-year follow-up (*n* = 244)	AD 2-year follow-up (*n* = 166)	Controls (*n* = 88)	*P* value*
Male patients, *n* (%)	173 (53.7%)	128 (52.4%)	86 (51.8)	54 (61.4%)	0.19
Age (years)	80.4 (6.2)	80.3 (6.2)	79.6 (6.5)	77.0 (6.2)	<0.01
Education (years)	10.1 (4.4)			10.1 (5.1)	0.96
Disease duration (months)	52.4 (47.5)			–	–
BMI (kg/m^2^)	23.6 (3.4)	23.7 (3.3)	23.9 (3.3)	24.4 (3.2)	0.07
MMSE	18.1 (5.9)	16.7 (6.4)	16.5 (6.6)	27.2 (2.5)	<0.01
Delayed recall of 12-item memory test	1.1 (1.8)				
Forward digit span	8.5 (3.2)				
Backward digit span	3.9 (2.1)				
Category verbal fluency	6.1 (3.0)				
Modified Boston naming test	11.1 (3.1)				
CDR, *n* (%)					
0.5	20 (6.2%)	10 (4.1%)	6 (3.6%)		
1	208 (64.6%)	129 (52.9%)	74 (44.6%)		
2	81 (25.2%)	81 (33.2%)	65 (39.2%)		
3	12 (3.7%)	23 (9.4%)	19 (11.4%)		
NPI-agitation/aggression	7.0 (12.0)	7.9 (12.5)	6.9 (11.4)		
NPI-mood	5.4 (9.4)	6.4 (9.9)	5.3 (8.6)		
NPI-frontal	5.7 (10.1)	7.2 (11.4)	5.7 (8.4)		
ApoE4 carrier, *n* (%)	135 (41.9%)			9 (10.2%)	< 0.01
Plasma clusterin level (μg/ml)	248.6 (37.9)			243.8 (35.9)	0.29
Aβ1–40 (pg/ml)	157.9 (50.7)			144.5 (36.9)	0.02
Aβ1–42 (pg/ml)	36.9 (20.8)			34.1 (9.1)	0.23
Ratio of Aβ1–42/Aβ1–40 (%)	23.7 (8.7)			24.4 (6.7)	0.32

Values are shown as means and standard deviations unless otherwise indicated

*Comparison between AD baseline and controls by Independent two sample *t* tests or Chi-square tests

*Aβ* amyloid beta, *AD* Alzheimer’s disease, *ApoE* apolipoprotein E, *BMI* body mass index, *CDR* Clinical Dementia Rating, *MMSE* Mini-Mental State Examination, *NPI* Neuropsychiatric Inventory

### Clinical evaluation and procedures

Cognitive function was assessed at baseline using standard procedures. The Mini-Mental State Examination (MMSE) was used to assess global cognition [28]. Clinical Dementia Rating (CDR) scores were used to represent the severity of dementia [29, 30]. We also performed a memory test using the delayed recall of 12 items [31, 32], a modified Boston naming test [33], and a category verbal fluency test to assess short-term memory and language function [34]. Forward and backward digit-span tests were used to assess attention and working memory [35]. The NPI questionnaire was administered to assess the frequency and severity of BPSD [1, 2]. The NPI agitation/aggression, mood, and frontal symptoms subscales were used in this study [3]. The NPI agitation/aggression subscale includes agitation/aggression, disinhibition, irritability, and aberrant motor behavior items. The NPI mood subscale includes depression, anxiety, and irritability items. The NPI frontal subscale includes apathy, disinhibition, irritability, and euphoria items. Each subscale score was calculated by summing the scores for the severity of each item and then multiplying the summed score by the duration. Follow-up examinations for AD patients were performed at 1 and 2 years. At each follow-up, we re-evaluated patient cognition, BPSD, and disease severity using the MMSE, NPI, and CDR scores. All tests were administered by trained individuals in a clinical setting.

### DNA and plasma clusterin level analyses

Blood samples were collected in tubes containing sodium EDTA as an anticoagulant. Following centrifugation, plasma was aliquoted into polypropylene tubes and stored at –80 °C until analysis. Genomic DNA was isolated from whole blood using the Gentra Puregene kit according to the manufacturer’s protocols (Qiagen, Hilden, Germany). The presence of the ε2, ε3, and ε4 alleles of the apolipoprotein gene (ApoE) was determined by assessing the sequences at two single-nucleotide polymorphisms (SNPs; rs429358 and rs7412) [36, 37]. An ApoE4 gene carrier was defined as having at least one allele containing the ε4 gene. The plasma Aβ peptide assay was performed using the INNO-BIA plasma Aβ forms assay (Innogenetics, Ghent, Belgium), which is based on the multiplex xMAP technique, with a LABScan-100 system (Luminex BV, Netherlands). The ratio of plasma Aβ1–42/Aβ1–40 was calculated as the plasma Aβ1–42 level divided by the plasma Aβ1–40 level. There was a significant difference in the ratio of plasma Aβ1–42/Aβ1–40 between patients with AD and controls [38]. Each sample was assessed in duplicate. The plasma clusterin levels were measured using a Human Clusterin Quantikine ELISA Kit (R&D Systems, Minneapolis, MN, USA) according to the manufacturer’s instructions. All samples from each participant were measured on the same plate to avoid interplate variation. The average intra-assay coefficient of variation based on duplicate samples was 1.2% for clusterin, 7.6% for Aβ1–40, and 9.1% for Aβ1–42. The average inter-assay coefficient of variation, as determined using three samples (low, medium, and high measurement range) that were analyzed on 4 different days, was 3.3% for clusterin, 12.0% for Aβ1–40, and 15.8% for Aβ1–42.

### Statistical analysis

All statistical analyses were performed using SPSS (version 21.0). Continuous variables are expressed as the means ± standard deviations. Categorical variables are presented as numbers and ratios. Independent *t* tests were performed to compare the ages, years of education, BMI, and MMSE scores of patients. Chi-square tests were used to compare AD patients and controls in terms of gender and ApoE4 carrier status. The analysis of variance (ANOVA) test and Kruskal-Wallis test were used to compare MMSE and NPI subscale scores with different levels of AD severity. We classified baseline plasma clusterin and Aβ levels into tertiles (low, median, and high) to study mean changes in MMSE scores at the 1-year follow-up (MMSE at 1-year subtracted from MMSE at baseline). One-way ANOVA tests were used to compare group differences. To investigate the relationship between plasma biomarkers and MMSE and NPI subscale scores during longitudinal follow-up, the generalized estimating equation (GEE) method was used to analyze longitudinally correlated data. Baseline plasma clusterin levels and the plasma Aβ1–42/Aβ1–40 ratio in patients with AD were then stratified into tertiles (low, median, and high). Analyses were performed using the GEE method because it accounts for variations in time and identifies correlations among repeat measurements that are collected during a longitudinal study design. In addition, the MMSE and NPI subscale scores did not display a normal distribution. For the GEE analyses, we used a robust estimator for the covariance matrix and selected autoregressive (1) for the working correlation matrix. Age, years of education, gender, and ApoE4 gene status were used to adjust the MMSE and NPI subscale scores. Interactions between groups, time, and ApoE4 carrier status were also analyzed by GEE. Estimated β values with 95% confidence intervals (CIs) were calculated for all the blood biomarkers. Statistical significance was defined as *p* < 0.05.

## Results

### Demographic and clinical data

In total, 322 AD patients (173 males and 149 females; mean age 80.4 ± 6.2 years; mean education 10.1 ± 4.4 years) and 88 controls (54 males and 34 females; mean age = 77.0 ± 6.2 years; mean education = 10.1 ± 5.1 years) were recruited. Controls were significantly younger than patients in the AD group (*p* < 0.01). There were no significant differences between the groups in terms of gender or years of education. MMSE scores differed significantly between patients with AD and controls (Table 1; *p* < 0.01). Compared with controls, a significantly higher proportion patients with AD were ApoE4 carriers (*p* < 0.01). The plasma Aβ1–40 levels were significantly higher in patients with AD compared with controls (Table 1; *p* = 0.02). The plasma Aβ1–42 and clusterin levels were similar between groups (*p* = 0.23 and 0.29, respectively). To study the differences in the plasma Aβ and clusterin levels between patients with AD and controls, regression analyses were performed to adjust for the effects of age, gender, ApoE4 carrier status, and years of education. Our results showed no significant differences between groups in the plasma Aβ1–40 or Aβ1–42, the Aβ1–42/Aβ1–40 ratio, or clusterin levels after correcting for multiple covariates (*p* = 0.15, 0.17, 0.69, and 0.29, respectively).

### Baseline characteristics for the complete study groups and the missing data group

Ultimately, 244 AD patients (75.8%) completed the 1-year clinical follow-up; 166 AD patients (51.5%) completed the 2-year clinical follow-up. At the 1-year follow up, the baseline characteristics (age, gender, years of education, ApoE4 carrier status, MMSE scores, CDR stage, NPI subscale scores, plasma clusterin, and plasma Aβ levels) were similar in both groups. At the 2-year follow-up, patients who did not complete the study were significantly older than those who participated in follow-ups (81.2 ± 5.8 vs. 79.6 ± 6.5 years, *p* = 0.02) and had lower MMSE scores (17.2 ± 6.1 vs. 19.2 ± 5.2, *p* < 0.01). There was no significant difference in ApoE4 carrier status, CDR stage, NPI subscale scores, plasma clusterin level, or Aβ level between the groups.

### Plasma biomarkers and dementia severity

To clarify the relationship between plasma biomarkers and disease severity, we stratified patients based on the severity of AD, as determined by the CDR scores. No significant differences in age, gender, or years of education were found among patients with different levels of AD severity. One-way ANOVA revealed no difference between the groups in terms of plasma clusterin, Aβ1–42 levels, and the Aβ1–42/Aβ1–40 ratio (*p* = 0.15, 0.51, and 0.08, respectively). There was a significant difference between the groups in the plasma Aβ1–42 levels (one-way ANOVA, *p* < 0.01), and post-hoc analysis showed that the plasma Aβ1–42 levels were significantly higher in the CDR = 3 group than in the CDR = 1 or 2 group (*p* < 0.05; Table 2).

**Table 2:** Demographic data of the patients with AD at baseline by disease severity

	CDR = 0.5 (*n* = 20)	CDR = 1.0 (*n* = 208)	CDR = 2.0 (*n* = 81)	CDR = 3.0 (*n* = 12)	*P* value*
Male patients, *n* (%)	13 (65.0%)	104 (50.0%)	49 (60.5%)	7 (58.3%)	0.29
Age (years)	79.2 (5.4)	80.3 (6.0)	80.6 (6.7)	82.5 (7.7)	0.49
Education (years)	9.6 (4.9)	10.2 (4.5)	9.7 (4.3)	12.4 (3.6)	0.22
Disease duration (months)	24.8 (16.7)	51.1 (48.6)	56.4 (39.7)	99.3 (71.6)	< 0.01
BMI (kg/m^2^)	24.5 (3.4)	23.6 (3.2)	23.9 (3.5)	20.3 (3.1)	0.03
MMSE	22.8 (2.9)	19.9 (4.2)	14.2 (5.0)	3.7 (3.5)	< 0.01
NPI-agitation/aggression	5.5 (13.3)	5.5 (10.0)	10.1 (13.5)	14.8 (22.1)	< 0.01
NPI-mood	6.3 (12.9)	4.7 (8.6)	6.5 (8.8)	9.8 (16.2)	0.15
NPI-frontal	5.1 (10.1)	4.6 (8.9)	8.0 (10.5)	11.3 (19.5)	0.01
ApoE4 carrier	6 (30%)	77 (37.0%)	44 (54.3%)	7 (58.3%)	< 0.01
Plasma clusterin level (μg/ml)	253.6 (36.9)	244.9 (36.6)	255.9 (40.9)	252.7 (38.9)	0.15
Aβ1–40 (pg/ml)	144.7 (28.7)	160.4 (47.8)	154.7 (55.5)	162.8 (87.2)	0.51
Aβ1–42 (pg/ml)	38.9 (11.9)	34.1 (13.9)	33.6 (15.6)	57.4 (87.4)	< 0.01
Ratio of Aβ1–42/Aβ1–40 (%)	26.9 (5.7)	21.8 (9.2)	22.5 (10.8)	28.3 (17.8)	0.08

Values are means with standard deviations unless otherwise indicated

*ANOVA or Kruskal-Wallis tests

*Aβ* amyloid beta, *AD* Alzheimer’s disease, *ApoE* apolipoprotein E, *BMI* body mass index, *CDR* Clinical Dementia Rating, *MMSE* Mini-Mental State Examination, *NPI* Neuropsychiatric Inventory

### Baseline plasma biomarkers and ApoE4 carrier status in AD

The Kruskal-Wallis test revealed that patients with severe AD were significantly more likely to carry the ApoE4 allele (Table 2; *p* < 0.01). Among the patients who were categorized as ApoE4 positive or negative, there was no significant difference in plasma levels of Aβ1–40, Aβ1–42, or the Aβ1–42/Aβ1–40 ratio. However, the plasma clusterin levels were significantly higher in the ApoE4-positive group (254.4 ± 33.8 μg/ml vs. 244.4 ± 40.2 μg/ml, *p* = 0.02). ApoE4 carrier status was used as a covariate in the longitudinal follow-up study.

### Plasma clusterin and Aβ levels at baseline and the 1-year follow-up

Based on the baseline plasma clusterin levels, we stratified patients into high (mean = 289.4 ± 22.6 μg/ml), medium (mean = 246.9 ± 8.1 μg/ml), and low (mean = 209.3 ± 23.4 μg/ml) tertiles to determine whether there was an association with baseline MMSE scores by one-way ANOVA. There was a significant group difference in MMSE scores (mean MMSE scores were 17.5 ± 6.3 in the high tertile, 18.1 ± 5.6 in the medium tertile, and 19.4 ± 4.6 in the low tertile; *p* = 0.04). Post-hoc analysis showed a difference between the high and low tertiles (*p* < 0.05). We further studied mean changes in MMSE scores at the 1-year follow-up. A significant within-group difference was found by one-way ANOVA (mean changes in MMSE scores at 1 year were −2.5 ± 3.4 in the high tertile, −2.0 ± 4.5 in the medium tertile, and −0.9 ± 3.5 in the low tertile; *p* = 0.02). Post-hoc analysis showed a significant difference between the high and low tertiles (*p* = 0.008). This suggests an association between higher plasma clusterin levels and lower cognitive status in patients with AD. For the analysis of plasma Aβ, Aβ1–40, Aβ1–42, and the Aβ1–42/Aβ1–40 ratio, patients were stratified into tertiles and we compared the mean changes in the MMSE scores at the 1-year follow-up using a one-way ANOVA. There were no significant differences in mean changes in MMSE scores among the tertiles of Aβ1–40, Aβ1–42, or the Aβ1–42/Aβ1–40 ratio (*p* = 0.77, 0.15, and 0.43, respectively).

### Plasma clusterin levels at the 2-year follow-up

**Fig. 1:**
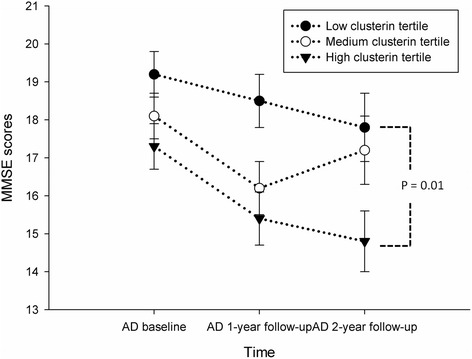
Plasma biomarkers associated with longitudinal changes in MMSE scores. Patients in the high tertile of plasma clusterin levels had significantly lower Mini-Mental State Examination (*MMSE*) scores compared with those in the low tertile based on a GEE analysis adjusted for age, gender, years of education, and ApoE4 carrier status (*p* = 0.01). There was no significant interaction between time and the tertile of plasma clusterin (*p* = 0.10). Data are presented as the mean ± standard error. *AD* Alzheimer’s disease

Longitudinal data collected over 2 years were analyzed by GEE adjusted for age, years of education, gender, time, and ApoE4 carrier status. This revealed that the MMSE scores were significantly decreased in patients in the high tertile of plasma clusterin levels (average decrease in MMSE scores after 2 years was −2.09 points, 95% CI = −3.67 to −0.51, *p* = 0.01) compared with patients in the low tertile (Fig. 1). This MMSE difference was the same difference as observed at baseline. No significant interactions were detected between the tertiles of plasma clusterin and time or between ApoE4 status and time (*p* = 0.10 and *p* = 0.16, respectively). MMSE scores were significantly lower among patients carrying the ApoE4 allele than those without (−2.75, 95% CI = −4.11 to −1.39, *p* < 0.01). Because plasma clusterin was significantly higher in the ApoE4-positive group, we performed an interaction analysis. No significant interaction between clusterin tertile and ApoE4 carrier status was detected (*p* = 0.93; Table 3). The plasma clusterin levels showed no association with agitation/aggression, mood, or frontal subscale NPI scores.

**Table 3:** Results of the generalized estimating equation analyzing the effect of 2-year MMSE changes in patients with AD

	β	SE	95% CI	*P* value
Gender				
Male	−0.32	0.83	(−1.95, 1.32)	0.71
Female	Referent			
Clusterin level				
High	−2.09	0.81	(−3.67, −0.51)	0.01
Medium	−0.87	0.71	(−2.27, 0.53)	0.22
Low	Referent			
ApoE4 carrier				
Positive	−2.75	0.69	(−4.11, −1.39)	0.00
Negative	Referent			
Age	0.03	0.08	(−0.12, 0.17)	0.74
Education years	0.13	0.09	(−0.04, 0.30)	0.15
Time	−0.91	0.21	(−1.33, −0.49)	0.00

Adjusted for age, education years, gender, time, and ApoE4 carrier

Interaction in group*ApoE4 carrier: *p* value = 0.93 and group*time: *p* value = 0.21

*AD* Alzheimer’s disease, *ApoE* apolipoprotein E, *CI* confidence interval, *MMSE* Mini-Mental State Examination, *SE* standard error

### Longitudinal analysis of plasma Aβ

**Fig. 2:**
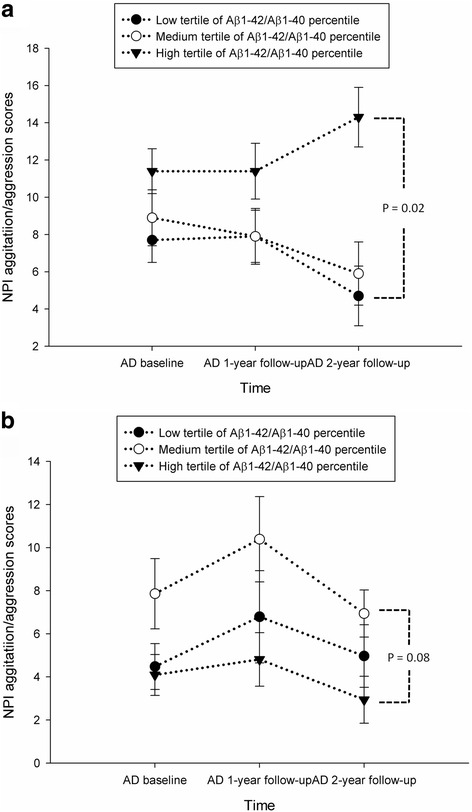
Plasma biomarkers associated with longitudinal changes in NPI-agitation/aggression scores based on the differences in ApoE4 carrier status. **a** Compared with the Aβ1–42/Aβ1–40 ratios in the low tertile, those in the high tertile were associated with a significant increase in NPI-agitation/aggression scores in ApoE4-positive carriers based on a GEE analysis adjusted for age, gender, and years of education (*p* = 0.02). **b** There was no significant difference between groups for NPI-agitation/aggression scores in ApoE4-negative carriers based on a GEE analysis adjusted for age, gender, and years of education. Data are presented as the mean ± standard error. *Aβ* amyloid beta peptide, *AD* Alzheimer’s disease, *NPI* Neuropsychiatric Inventory

To explore the predictive value of plasma Aβ for cognition and BPSD, we stratified patients by the plasma Aβ1–42/Aβ1–40 ratio into high (mean = 31.9 ± 10.2%), medium (mean = 22.6 ± 1.4%), or low (mean = 16.7 ± 2.5%) groups. After adjustment for age, years of education, gender, time, and ApoE4 carrier status, GEE analysis revealed no association between the plasma Aβ1–42/Aβ1–40 ratio and the decline in the MMSE score at 2 years (*p* = 0.12). GEE analysis showed that the plasma Aβ1–42/Aβ1–40 ratio and ApoE4 carrier status were significant predictors of the NPI agitation/aggression score (*p* = 0.04 and 0.009, respectively). GEE analysis also revealed a significant interaction between the plasma Aβ1–42/Aβ1–40 ratio and ApoE4 carrier status (*p* = 0.003). Next, we stratified patients with AD based on ApoE4 carrier status (135 ApoE4 carriers and 187 non-carriers). After 2 years, ApoE4 carriers in the high plasma Aβ1–42/Aβ1–40 ratio tertile showed a significant increase in the NPI agitation/aggression score compared with those in the low tertile (6.06, 95% CI = 1.20–10.62, *p* = 0.02; Table 4, Fig. 2a). No such association was found among ApoE4-negative patients (Fig. 2b). The plasma Aβ1–42/Aβ1–40 ratio was not a significant predictor of NPI-mood or NPI-frontal scores. GEE analysis revealed no significant association between plasma Aβ1–42 level and MMSE or NPI subscale scores at 2 years.

**Table 4:** Results of the generalized estimating equation analyzing the effect of 2-year NPI agitation/aggression changes in ApoE4-positive patients with AD

	β	SE	95% CI	*P* value
Gender				
Male	0.16	2.25	(−4.25, 4.57)	0.94
Female	Ref			
Ratio of Aβ1–42/Aβ1–40				
High	6.06	2.48	(1.20, 10.62)	0.02
Medium	1.20	2.24	(−3.18, 5.59)	0.59
Low	Ref			
Age	0.18	0.14	(−0.10, 0.46)	0.21
Education years	0.11	0.17	(−0.23, 0.45)	0.51
Time	−0.96	0.70	(−2.34, 0.42)	0.17

Adjusted for age, education years, gender, and time

*Aβ* amyloid beta peptide, *AD* Alzheimer’s disease, *CI* confidence interval, *NPI* Neuropsychiatric Inventory, *SE* standard error

## Discussion

This study investigated the utility of plasma clusterin and Aβ levels as prognostic markers in AD. Higher plasma clusterin levels were associated with significantly lower MMSE scores at baseline. At the 1- and 2-year follow-up, GEE analyses of AD patients revealed that high levels of plasma clusterin at baseline were associated with a significantly larger decrease in MMSE compared with low levels of plasma clusterin (after adjustment for multiple covariate factors). However, there was no significant interaction between time and the clusterin level tertile, which indicates that higher levels of plasma clusterin were not associated with the rapid decline of MMSE scores over time. The ApoE4 carrier status of patients with AD also displayed a similar pattern. Literature shows that ApoE4 and plasma clusterin are biomarkers for an accelerated cognitive decline in AD [39]. However, our results indicate that the plasma clusterin level is a biomarker for the severity of cognitive function decline in patients with AD rather than a prognostic marker.

The results of our longitudinal study on plasma Aβ levels showed no association with a change in cognition, which is consistent with previous findings [4, 40]. In the ApoE4-positive group, patients in the high but not the low tertile for plasma Aβ1–42/Aβ1–40 ratio showed a significant increase in NPI agitation/aggression scores at the 2-year follow-up. These findings indicate that the plasma Aβ1–42/Aβ1–40 ratio could serve as a biomarker for agitation/aggression symptoms during the long-term follow-up of ApoE4 carriers. To the best of our knowledge, this has not been proposed in previous studies.

### Plasma clusterin levels and cognitive decline

At present there is no reliable biomarker for predicting the speed of AD progression [41, 42]. The plasma level of clusterin could be a prognostic marker for patients with AD, but the specific relationship between the clusterin level and cognitive decline remains to be determined. A previous study reported that higher concentrations of plasma clusterin were significantly associated with an accelerated cognitive decline in patients with AD after 1 year of follow-up [23]. In patients with MCI, higher baseline levels of plasma clusterin have been associated with lower rates of brain atrophy. The authors of that study proposed that increased clusterin levels could protect at-risk subjects; failure of this mechanism in patients with AD would account for a more rapid decline in cognition and brain structural changes [43]. Another study reported conflicting results, showing that higher clusterin levels were related to less cognitive decline in patients with AD [22]. The results from our 2-year follow-up showed that higher levels of plasma clusterin were not associated with a more rapid cognitive decline in AD, which indicates that plasma clusterin levels are related to cognitive state and disease severity in AD [19, 44].

Many confounding factors may contribute to plasma clusterin levels. Recent studies have shown that plasma clusterin levels can be affected by gender, obesity, systemic inflammation, or atherogenic components of the lipid profile in AD patients [45, 46]. Plasma clusterin levels also play a role in tissue remodeling and immune responses in the brain, which may be related to the speed of deterioration in AD patients [45–48]. Further research is necessary to explore a potential role for clusterin in response to tissue damage and how it could influence the progression of AD. In practice, interpreting the plasma clusterin level is required to determine the disease state of a subject.

### Plasma Aβ predicts agitation/aggression symptoms in ApoE4 carriers

In our current work, we found that the plasma Aβ1–42/Aβ1–40 ratio at baseline could predict the increase in NPI agitation/aggression scores among ApoE4 carriers. Agitation and aggression symptoms are difficult to manage and are especially distressing for patients, caregivers, and medical staff members. In a previous study, the CSF level of Aβ1–42 was associated with agitation and irritability symptoms in patients with MCI [49]. β-Amyloid precursor protein, a precursor for Aβ, has also been linked to aggression symptoms in young children with autism [50]. The ApoE4 gene also contributes to aggression symptoms in patients with AD [51, 52]. Our findings suggest that plasma Aβ may act synergistically with the ApoE4 gene to aggravate agitation/aggression symptoms in patients with AD because the ApoE4-negative group did not show this association. Additional large, cohort studies with longitudinal follow-ups will be needed to confirm this finding.

### Plasma Aβ and cognitive changes in AD

Our study found no significant differences in the plasma Aβ levels between patients with AD and controls after adjustment for multiple covariates, nor was there a longitudinal association with MMSE changes in patients with AD. Previous studies have shown increased plasma Aβ1–40 or Aβ1–42 levels in patients with AD [7, 53], but negative studies have also been reported [54, 55]. Longitudinal studies on the association between the plasma Aβ levels and cognitive changes have also shown both positive [11, 56] and negative results [4]. This variation in studies on plasma Aβ may be the result of different collection methods [57], assay techniques [58], and multiple confounding factors such as diabetes, hypertension, or medication use [59, 60]. A recent study demonstrated an association between chemically treated plasma Aβ and the deposition of brain amyloid, which may provide a potential blood-based biomarker for the study of AD [61].

### Limitations

There are several limitations to the current study. Over the 2 years of follow-up, patients exhibited disease progression, cognitive decline, and an increased severity of neuropsychiatric symptoms, such as agitation and aggression [62]. This could be part of the natural course of the disease; MMSE and NPI symptoms associated with AD decline at different rates depending on the severity of AD. Although levels of plasma clusterin did not differ significantly among patients with different levels of AD severity, we cannot exclude such a possibility. Other confounding factors, such as depression and agitation symptoms, have been reported to increase the plasma clusterin levels, and the plasma amyloid levels may be affected by genetic variants and creatinine levels [47, 63, 64]. We acknowledge this limitation, and consider both plasma biomarkers to be predictors of clinical phenotype, rather than exhibiting a direct causal relationship. Second, we did not include other plasma biomarkers or imaging data in this study. CSF Aβ1–42 levels or brain amyloid scans could provide more reliable pathological markers than plasma biomarkers for the diagnosis of AD [65]. Measurements of structural changes in brain regions could be a robust marker, although it is more expensive than plasma biomarkers. Our previous cross-sectional study showed that the score for right-side posterior atrophy, as measured by brain MRI, was associated with NPI agitation/aggression symptoms [66]. A longitudinally repeated MRI measurement could be useful for linking plasma biomarkers with brain morphology and BPSD. Third, our drop-out rate at the 2-year follow-up was 49%, and the missing data group had an older age and lower MMSE scores than did the complete study group. Although there were no differences in plasma clusterin or Aβ levels between groups, we cannot exclude the possibility of bias caused by our missing data. A study with a larger sample size and a higher follow-up rate may improve our findings.

## Conclusions

In summary, our results suggest that the highest tertile of baseline plasma clusterin levels are associated with lower MMSE scores. The plasma clusterin level could be a marker of cognitive severity rather than a prognostic marker in patients with AD. A high plasma Aβ1–42/Aβ1–40 ratio combined with the ApoE4 gene may increase NPI agitation/aggression symptoms in patients with AD after 2 years of follow-up, which indicates a surrogate prognostic marker. These results will provide clinicians and caregivers with information on disease severity and progression in patients with AD, and give them a clear picture of the future to assist with designing a suitable care plan for patients with AD.

## Abbreviations

AD: Alzheimer’s disease
ANOVA: Analysis of variance
ApoE: Apolipoprotein E
Aβ: Beta-amyloid
BMI: Body mass index
BPSD: Behaviors and psychological symptoms of dementia
CDR: Clinical Dementia Rating
CI: Confidence interval
CSF: Cerebrospinal fluid
GEE: Generalized estimating equation
MCI: Mild cognitive impairment
MMSE: Mini-Mental State Examination
MRI: Magnetic resonance imaging
NPI: Neuropsychiatric Inventory
